# Supplementary material for: Mechanically interlocked functionalization of monoclonal antibodies
Source: Nat Commun. 2018 Apr 20;9:1580. doi: 10.1038/s41467-018-03976-5 (PMC5910394; doi:10.1038/s41467-018-03976-5)
Supplement: Supplementary file 1 — Supplementary Information [file 41467_2018_3976_MOESM1_ESM.pdf]

Supplementary Figures

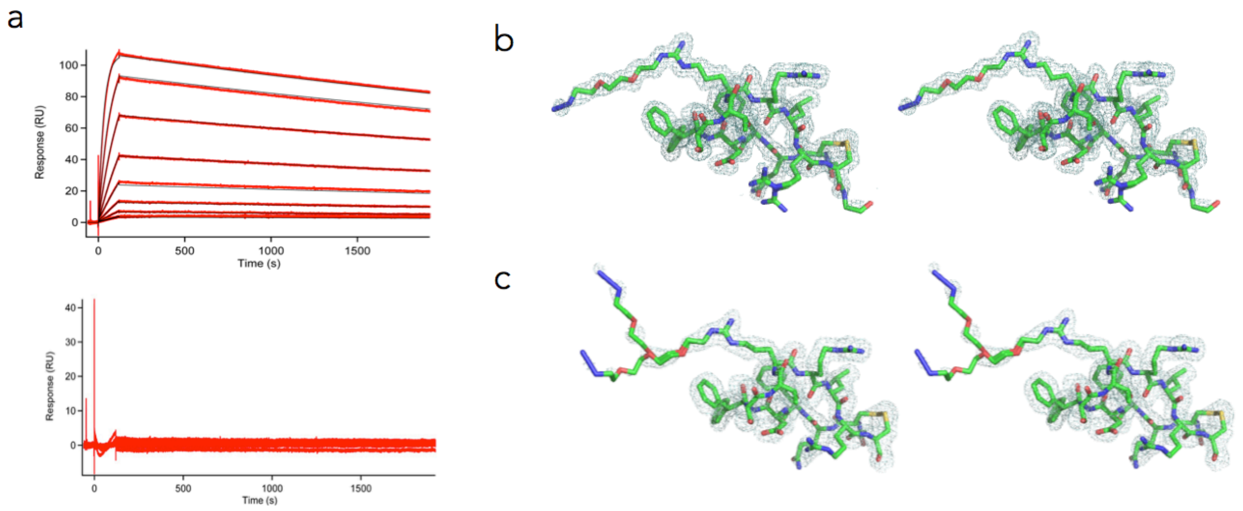

Supplementary Figure 1. a) Top - SPR sensogram showing the binding interaction of 5-diphenylalanine-8-Arg-$(PEG)_2$-azidomeditope with Ile83Glu memAb anti-HER2 IgG immobilized on the sensor chip, at 37 ºC (n=1). Analyte samples were prepared as a two-fold dilution series from 500 pM to 39 pM. Calculated K_D = 2.1 nM, $k_a = 6.6 \times 10^4\ M^{-1}s^{-1}$ and $k_d = 1.4 \times 10^{-4}\ s^{-1}$. Experimental data is in red, the corresponding fit is in black. Bottom – residuals plot obtained from fitting the experimental data to a 1:1 interaction model. b) Electron density omit map of 5-diphenylalanine-8-Arg-$(PEG)_2$-azidomeditope and c) 5-diphenylalanine-8-Arg-$(PEG)_3$-azidomeditope, in stereo and contoured at 0.8 σ .

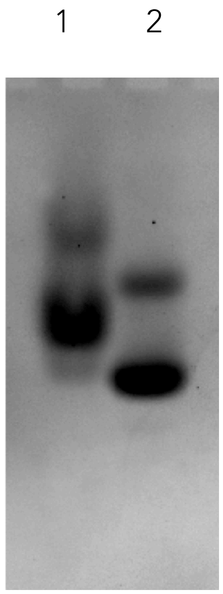

Supplementary Figure 2. Native PAGE of Ile83Glu memAb anti-HER2 Fab with locked-on 5-diphenylalanine-8-Arg-(PEG)$_3$-azidomeditope-DBCO-Cys-His$_6$ (lane 1) and Ile83Glu memAb anti-HER2 Fab (lane 2). Locked-on meditope was prepared as follows: Fab (60 µL, 0.3 mM) was mixed with 5-diphenylalanine-8-Arg-(PEG)$_3$-azidomeditope at a 1:1 molar ratio and incubated for 30 min at 25 °C prior to the addition of 5.5 molar excess of DBCO-Cys-His$_6$ (10 µL, 10 mM). After 2.5 h, the product was purified by Ni-NTA affinity chromatography and unreacted DBCO-Cys-His$_6$ was removed on the Zeba spin column.

a

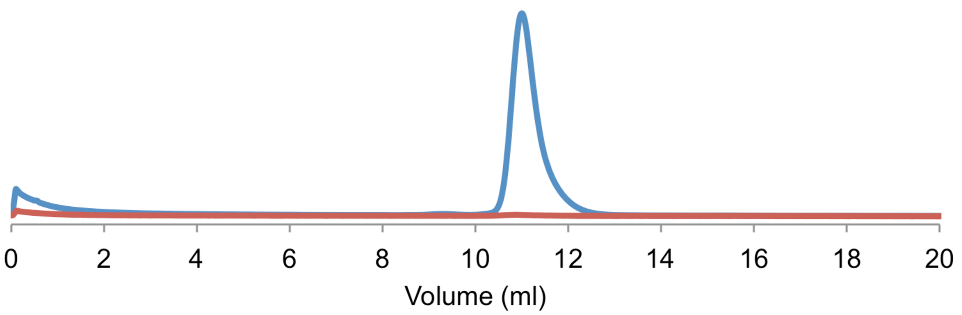

b

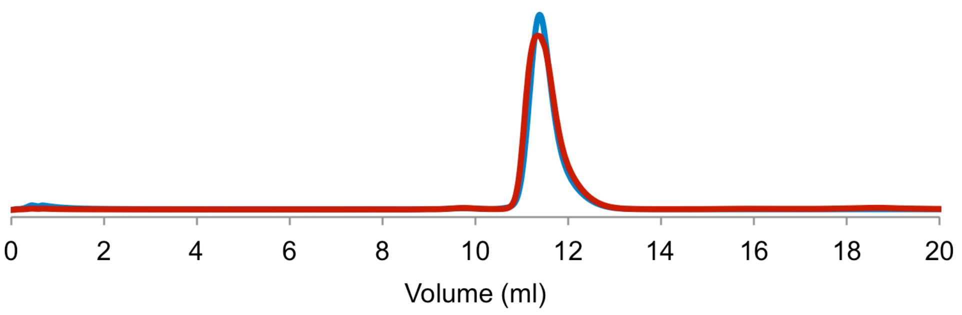

Supplementary Figure 3. Purification of locked on meditope-AF647 I83E memAb anti-HER2 Fab complex. a) SEC traces of reaction product between Ile83Glu memAb anti-HER2 Fab and DIBO-AF647 (no meditope added), after purification on Zeba spin column. Lack of absorbance at 650 nm (red trace) in the elution peak of the Fab (280 nm, red trace) indicates no non-specific binding of DIBO-AF647 to the Fab. b) SEC traces of reaction product between Ile83Glu memAb anti-HER2 Fab-5-diphenylalanine-8-Arg-$(PEG)_3$-azidomeditope and DIBO-AF647 purified on Zeba spin column. Co-elution of the Fab (280 nm, blue trace) and AF647 (at 650 nm, red trace) indicates that the click reaction was successful. Fractions containing locked-on meditope-AF647-Fab complex were used for stability experiments (below).

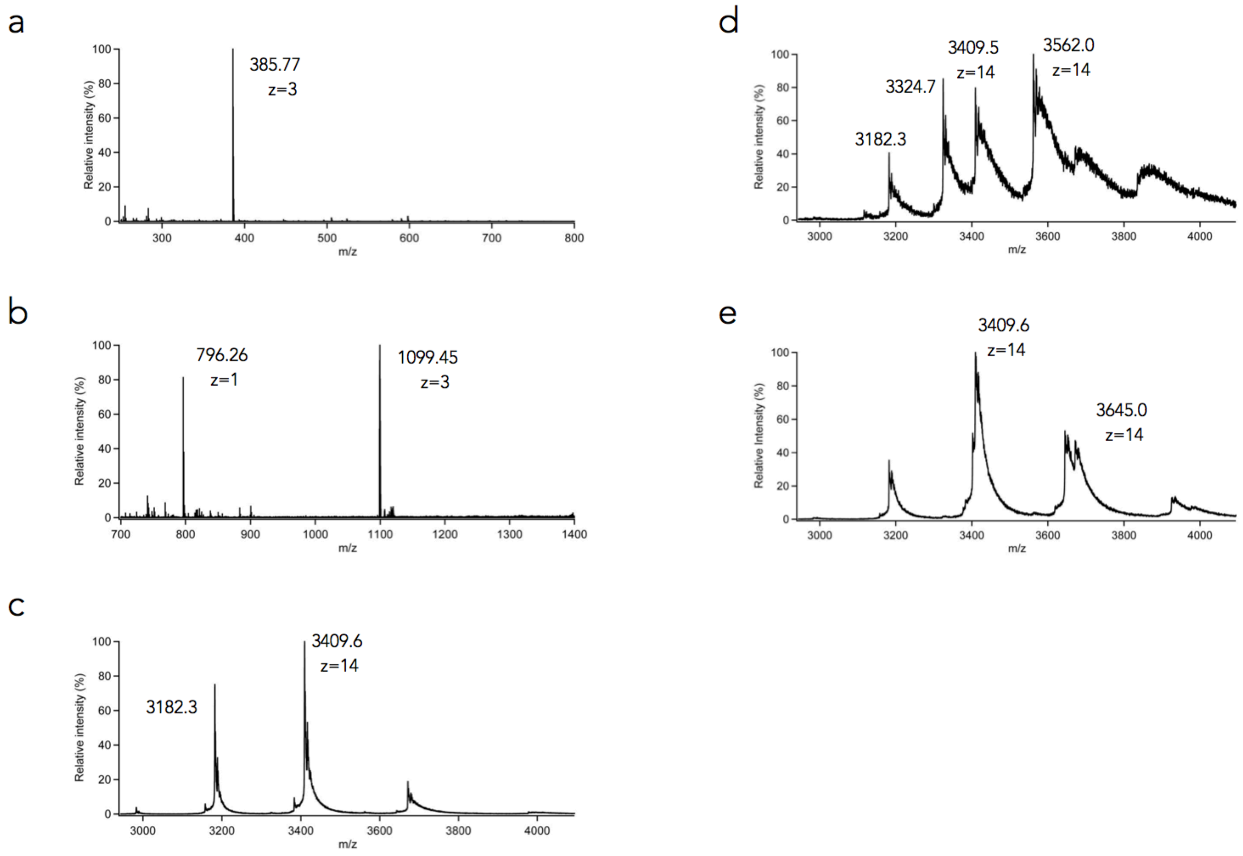

Supplementary Figure 4. Mass spectrometry analysis. a) DIBO-AF647 (negative mode measured, MW 1160.33 – note the exact structure is unknown, Life Technologies lists the MW as ~1500); b) 5-diphenylalanine-8-Arg-$(PEG)_2$-azidomeditope-DIBO-AF647 conjugate (positive ion, calculated MW 3293.34, measured MW 3293.32); c) Ile83Glu memAb anti-HER2 Fab (positive ion, calculated MW 47712, measured MW 47720), d) Ile83Glu memAb anti-HER2 Fab with 5-diphenylalanine-8-Arg-$(PEG)_2$-azidomeditope (positive ion, calculated MW 49846, measured MW 49854), and e) Ile83Glu memAb anti-HER2 Fab with locked-on 5-diphenylalanine-8-Arg-$(PEG)_2$-azidomeditope-DIBO-AF647 (positive ion, calculated MW 51005, measured MW 51017).

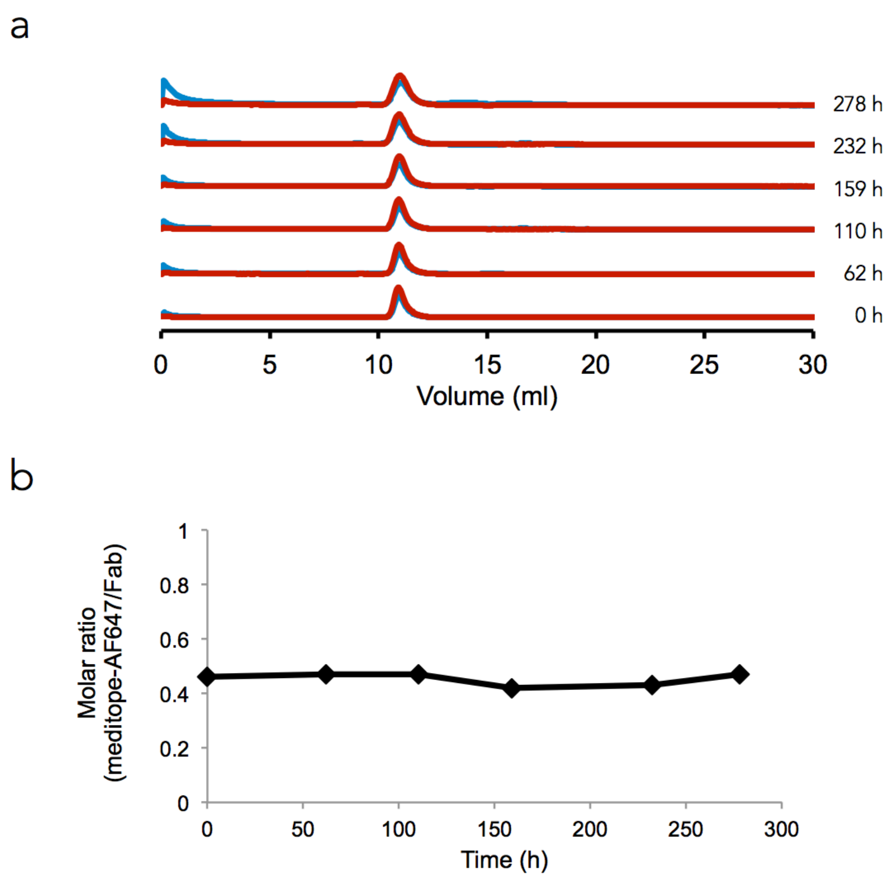

Supplementary Figure 5. a) Stability of the mechanically interlocked Ile83Glu memAb anti-HER2 Fab 5-diphenylalanine-8-Arg-$(PEG)_2$-azidomeditope-DIBO-AF647 complex – analysis by SEC. The complex (starting volume 150 µl) was dialyzed against PBS (500 ml) in a dialyzer with a MWCO 7 kDa membrane. Buffer was changed at 62, 86, 110, 134, 159 and 230 h. Samples were assayed by SEC (S75 10/300 Increase) at 0, 62, 110, 159, 232 and 278 h. Blue trace – absorbance at 280 nm corresponding to protein; red trace – absorbance at 650 nm corresponding to AlexaFluor647. Plots are normalized at each time point to maximum absorbance at 650 nm. The broadening of the peaks is the result of sample dilution during the course of the experiment. b) Molar ratio of Fab to 5-diphenylalanine-8-Arg-$(PEG)_3$-azidomeditope-DIBO-AF647 during dialysis.

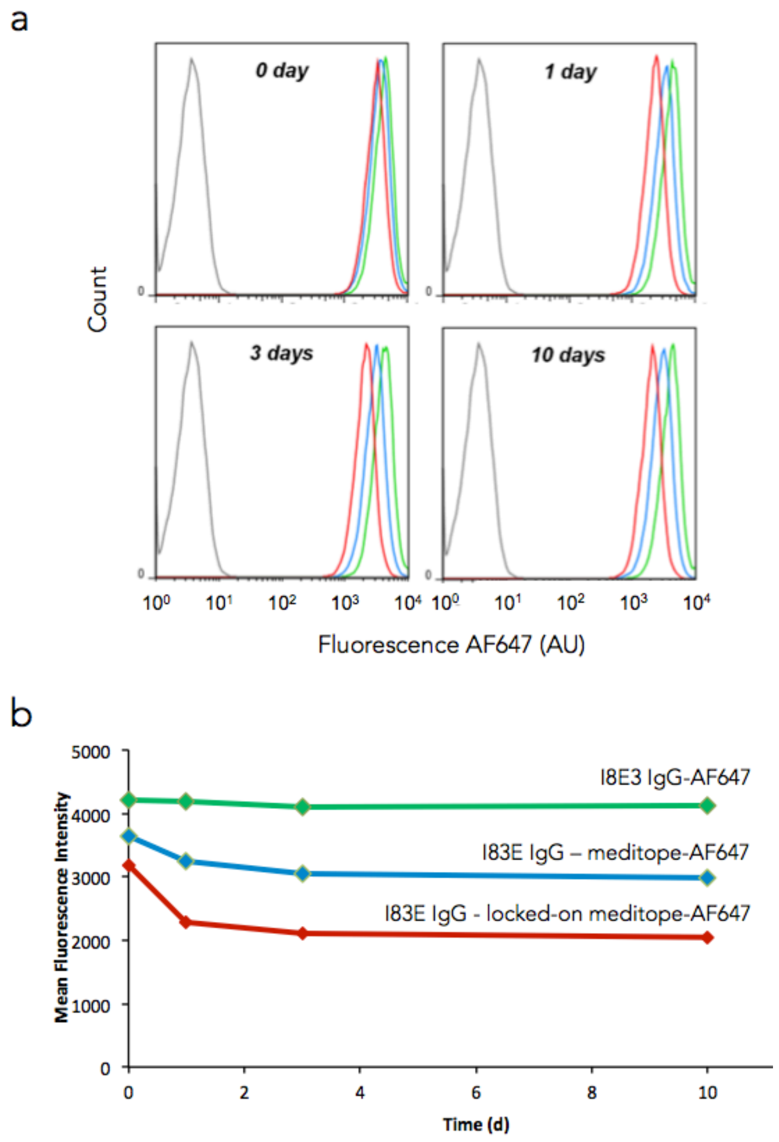

Supplementary Figure 6. Stability of the mechanically interlocked Ile83E memAb anti-HER2 IgG meditope-DIBO-AF647 complex. a) analysis by flow cytometry: gray – untreated BT474 cells, red – mechanical bond - Ile83Glu memAb anti-HER2 IgG complex, blue – AF647-5-diphenyl-meditope – I83E memAb anti-HER2 IgG complex, green – Ile83Glu memAb anti-HER2 IgG labeled with AF647. b) Plot of mean fluorescence vs. time for data presented in panel a) Color scheme is the same as in panel a).

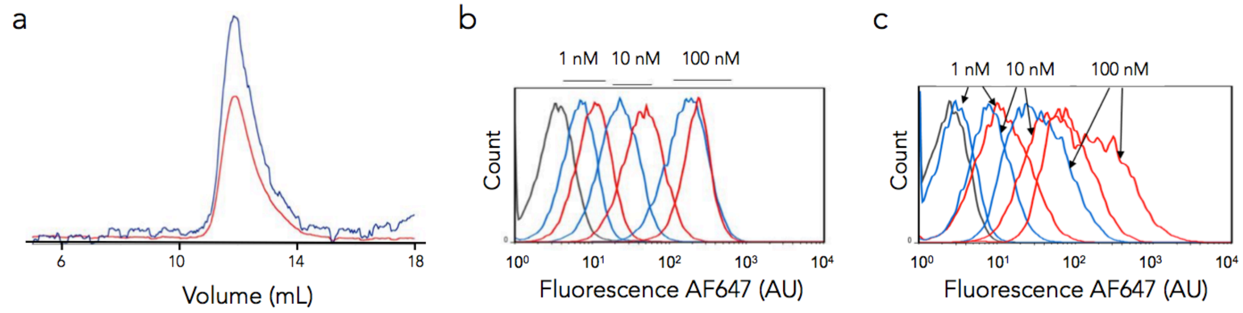

Supplementary Figure 7. Examples of mechanically interlocked antibodies. a) locked-on 5-diphenylalanine-8-Arg-$(PEG)_3$-azidomeditope-DIBO-AF647-memAb OKT3 IgG (α-CD3 antibody) purification (Sephadex S75 10/300; blue trace – absorbance at 650 nm corresponding to AlexaFluor647; red trace – absorbance at 280 nm). 150 µL of 100 µM memAb OKT3 IgG were incubated with 50 µL of 0.9 mM 5-diphenylalanine-8-Arg-$(PEG)_3$-azidomeditope for 10 min at room temperature. 20 µL of 8 mM DIBO-Alexa647 was added and the reaction was allowed to proceed for 16 h at room temperature. b) Binding of memAb pertuzumab IgG, an α-EGFR antibody, to BT474 cells: memAb pertuzumab IgG with pre-bound 5-diphenylalanine-meditope-Alexa-647 (blue) and locked-on 5-diphenylalanine-8-Arg-$(PEG)_3$-azidomeditope-DIBO-AF647-memAb pertuzumab IgG (red); black trace is for untreated cells. c) Binding of memAb M5A, an α-CEA antibody, to LS174T cells: memAb M5A IgG with pre-bound 5-diphenylalanine-meditope-Alexa-647 (blue) and locked-on 5-diphenylalanine-8-Arg-$(PEG)_3$-azidomeditope-DIBO-AF647-memAb M5A IgG (red); black trace is for untreated cells. Locked-on products were prepared as follows: 70 µL of the IgG (at 32 µM, pertuzumab IgG or 19 µM M5A IgG) were mixed with 3-fold molar excess of 5-diphenylalanine-8-Arg-$(PEG)_3$-azidomeditope. 5-fold molar excess of DIBO-Alexa647 was added 15 min later, and the reaction was allowed to proceed for 2h (M5A) and 3.5 h (pertuzumab) at room temperature followed by purification by SEC (Superdex 200 10/300).

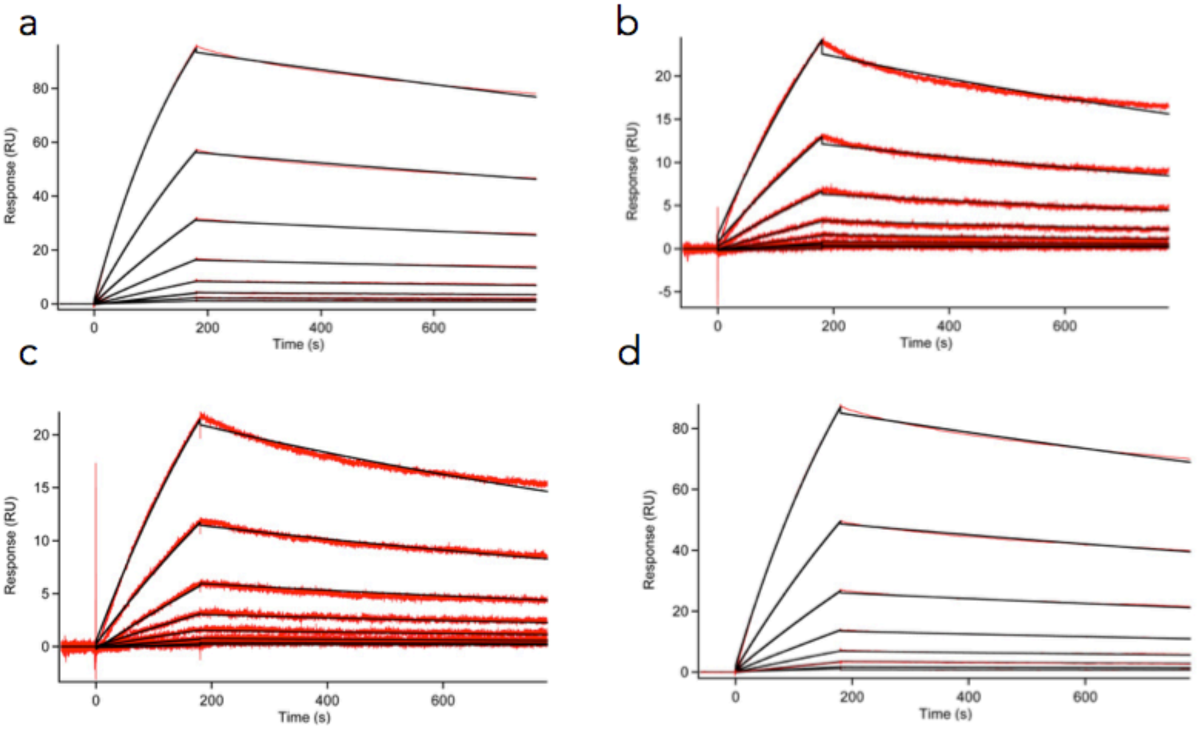

Supplementary Figure 8. Representative SPR sensograms of apo and holo Ile83Glu memAb anti-HER2 Fabs binding to HER2 at 37 °C. Analyte at 0.039 – 5 nM was flowed over HER2 immobilized on a Series S CM5 sensor chip. All experiments were done in triplicate. Data is presented as mean ± standard deviation. Experimental data (red) was fitted to 1:1 interaction model (black lines) as detailed in the methods section. a) Sensograms of Ile83Glu memAb anti-HER2 Fab binding to HER2. k_a=8.0±1.9x10^5 M^{-1}s^{-1}, k_d=6.0±2.1x10^{-4} s^{-1}, K_D=0.82±0.48 nM. b) Sensograms of Ile83Glu memAb anti-HER2 Fab with 50x molar excess of 5-diphenylalanine-8-Arg-(PEG)$_3$-azidomeditope binding to HER2. k_a=8.2±2.8x10^5 M^{-1}s^{-1}, k_d=7.9±0.8x10^{-4} s^{-1}, K_D= 1.1±0.3 nM. c) Sensograms of Ile83Glu memAb anti-HER2 Fab with locked on 5-diphenylalanine-8-Arg-(PEG)$_3$-azidomeditope –DIBO-AF647 binding to HER2. k_a=8.6±2.7x10^5 M^{-1}s^{-1}, k_d=7.0±1.8x10^{-4} s^{-1}, K_D= 0.84±0.11 nM . d) Sensograms of Ile83Glu memAb anti-HER2 Fab with locked on 5-diphenylalanine-8-Arg-(PEG)$_3$-azidomeditope–DBCO-Cys-His$_6$ binding to HER2. k_a=7.2±0.1x10^5 M^{-1}s^{-1}, k_d=3.6±0.1x10^{-4} s^{-1}, K_D= 0.50±0.01 nM .

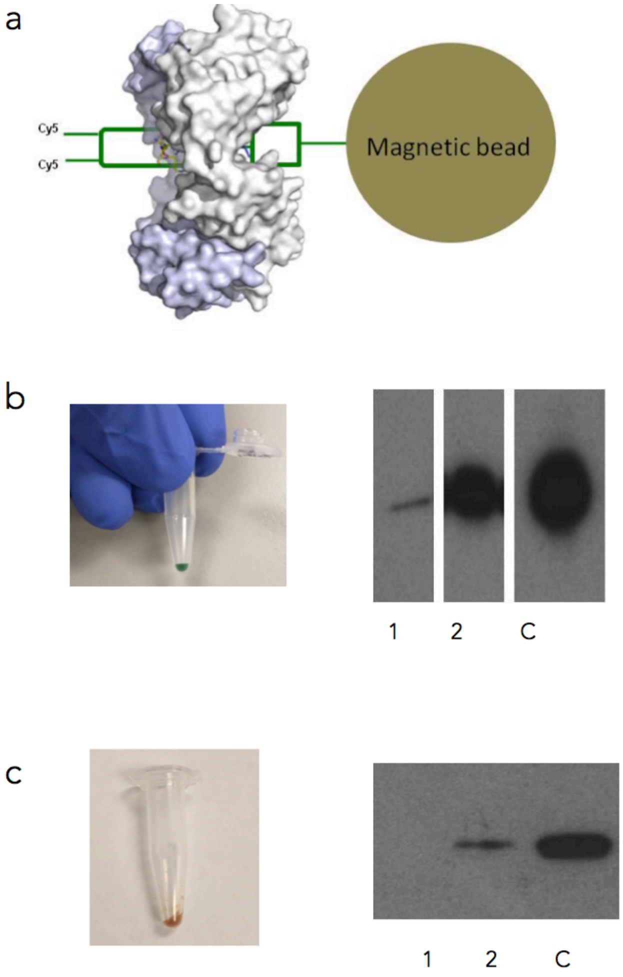

Supplementary Figure 9. Click chemistry product between 5-diphenylalanine-8-Arg-(PEG)$_2$-azidomeditope-(Cy5)$_2$/Ile83E memAb anti-HER2 Fab and DBCO-magnetic beads. a) Schematic representation of the reaction product. Only a single modification site on the bead is shown. b) Reaction products: locked-on meditope-DBCO magnetic beads/Fab (left) and corresponding Western blot with an α-κ light chain antibody-HRP (lane 1 – separated supernatant, lane 2 – Fab bound to beads, lane c – control Fab - 0.36 μg loaded). c) Determination of amount of non-specifically bound Fab – beads processed as in b) but no azidomeditope was present in the reaction mixture (left) and corresponding Western (1 – supernatant, 2 – Fab bound to beads, c – control Fab - 0.1 μg loaded). Based on densitometric analysis of the westerns with Image J, about 26% of the Fab in b) is non-specifically bound to the beads, with 74% locked-on with azidomeditope.

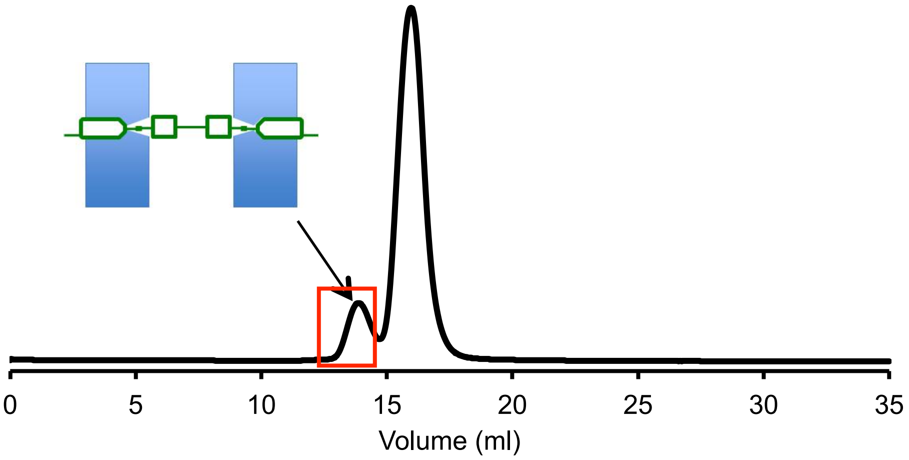

Supplementary Figure 10. Purification of a “molecular dumbbell” - reaction product between 5-diphenylalanine-8-Arg-$(PEG)_3$-azidomeditope/Ile83Glu memAb anti-HER2 Fab complex and DBCO-AcKSADASK-DBCO on Superdex 75 10/300. The peak corresponding to ~100 kDa (red box) was purified and analyzed by mass spectrometry.

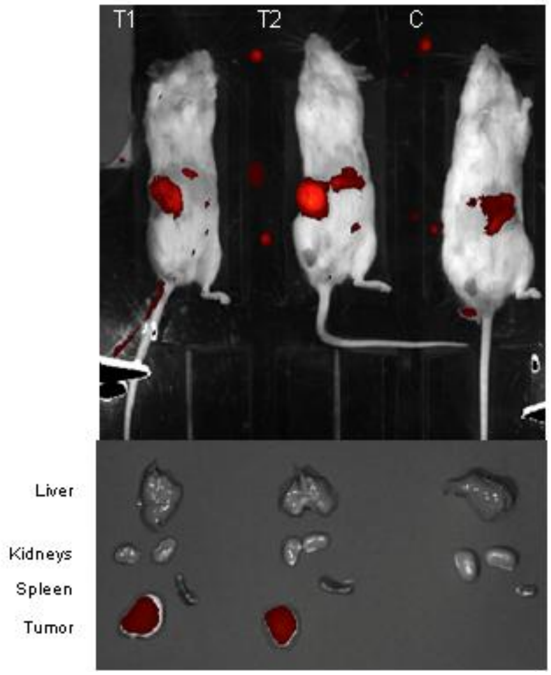

Supplementary Figure 11. Xenogen images of mice bearing BT474 xenograft tumors (top panel, mice T1 and T2) and injected through tail vain with mechanically interlocked-meditope-AF647-Ile83Glu memAb-anti-HER2 IgG. Images were taken 24 h post injection. C indicates the non-tumor bearing control mouse. Bottom panel: distribution of mechanically interlocked AF647-meditope-Ile83Glu memAb anti-HER2 in tumors and various organs 8 days post-injection.

Supplementary Table 1. Binding kinetics for different cyclic meditope variants to memAb anti-HER2 and Ile83Glu memAb anti-HER2 (n=1, except last three (*) which are n=4).

Meditope	k_a ($M^{-1}s^{-1}$) x 10^4	k_d (s^{-1})	K_D (nM)
Binding kinetics to memAb anti-HER2 at 25°C			
CQFDLSTRRLKC	1.3	0.016	1200
(Ac)CQFDLSTRRLRCGGSK	2.8	0.006	210
(MPT) QFDLSTRRLKC[1]	1.8	0.018	1000
GQFDLSTRRLKG[1]	1.9	0.12	6300
(AHA)QFDLSTRRLK[1]	2.1	0.12	5700
CQ(4-Br)FDLSTRRLKC	0.91	0.042	4700
CQYNLSSRALKC	0.97	0.033	3400
CQYDLSTRRLKC	2.8	0.11	3900
CQA$(Ph)_2$DLSTRRLKC	0.92	0.024	2600
CQFDA$(Ph)_2$STRRLKC	20	0.008	40
(Ac)CQFDA$(Ph)_2$STRRLRCGGSK	5.6	0.0023	40
Binding kinetics to memAb anti-HER2 at 37 °C			
CQFDLSTRRLKC	4.4	0.044	1000
(Ac)CQFDLSTRRLRCGGSK	7.5	0.026	350
(Ac)CQFDA$(Ph)_2$STRRLRCGGSK	28	0.011	39
Binding kinetics to Ile83Glu memAb anti-HER2 at 25°C			
CQFDLSTRRLKC	2.6	5.9×10^{-4}	23
(Ac)CQFDLSTRRLRCGGSK	71	3.1×10^{-4}	0.44
(Ac)CQFDA$(Ph)_2$STRRLRCGGSK			0.026[2]
*Binding kinetics to Ile83Glu memAb anti-HER2 at 37°C			
CQFDLSTRRLKC	26 (±25)	25 (±0.3) $\times 10^{-4}$	20 (±2)
(Ac)CQFDLSTRRLRCGGSK	16 (±6)	6.3 (±0.5) $\times 10^{-4}$	4.0 (±0.8)
(Ac)CQFDA$(Ph)_2$STRRLRCGGSK	71 (±61)	2.2 (±1) $\times 10^{-4}$	0.40 (±0.2)

[1] (MPT) QFDLSTRRLKC – mercaptopropionic acid – cysteine linker; GQFDLSTRRLKG -di-glycine linker; (AHA)QFDLSTRRLK – aminoheptanoic acid linker.

[2] estimated value based on k_a of 38×10^5 $M^{-1}s^{-1}$ and k_d of 1×10^{-5} s^{-1} (instrument's limit).

Supplementary methods

Synthesis of azido-meditope variants

Synthetic precursors of azido-meditope variants a and b (below)

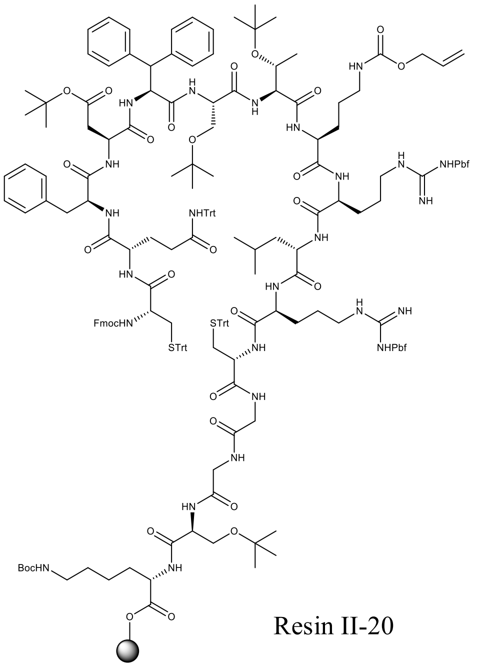

Resin II-20

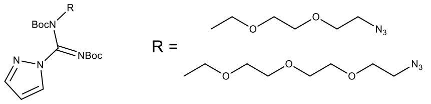

a

b

Structures of 5-diphenylalanine-8-Arg-(PEG)$_2$-azidomeditope (azido-meditope variant a, below left) and 5-diphenylalanine-8-Arg-(PEG)$_3$-azidomeditope (azido-meditope variant b, below right)

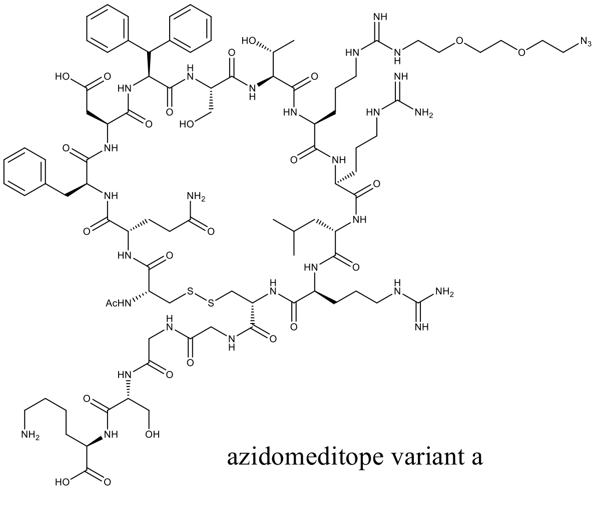

azidomeditope variant a

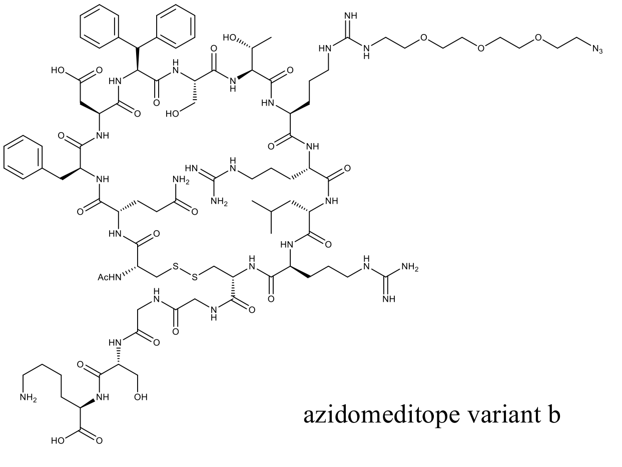

azidomeditope variant b

Amino acid sequences of IgGs used in this study

Meditope-enabled OKT3 amino acid sequence:

Light Chain (LC):

QIVLTQSPIIMSASPGEKVTMTCSASSSVSYMNWYQQRTNGSPRRWIYDTSKLASGVPA
HFRGSGSGTSYSLTISGMEAEDAADYYCQQWSSNPFTFGSGTKLEINRADTAPTVSIFPPS
SEQLTSGGASVVCFLNNFYPKDINVKWKIDGSERQNGVLNSWTEQDSKDSTYSMSSTLT
LTKDEYERHNSYTCEATHKTSTSPIVKSFNRNEC*

Heavy Chain (HC):

QVQLQQSGGELARPGASVKMSCKASGYTFTRYTMHWVKQSPGKGLEWIGYINPSRGY
TNYNQKFKDKATLTTDKSSSTAYMQLSSLTSEDSAIYYCARYYDDHYCLDYWGQGTLL
TVSSAKTTAPSVYPLAPVCGGTTGSSVTLGCLVKGYFPEPVTLTWNSGSLSSGVHTFPCV
LQSDLYTLSSSVTVTSSTWPSQSITCNVAHPASSTKVDKKIEPRGPTIKPCPPCKCPAPNLL
GGPSVFIFPPKIKDVLMISLSPIVTCVVVDVSEDDPDVQISWFVNNVEVHTAQTQTHRED
YNSTLRVVSALPIQHQDWMSGKEFKCKVNNKDLPAPIERTISKPKGSVRAPQVYVLPPP
EEEMTKKQVTLTCMVTDFMPEDIYVEWTNNGKTELNYKNTEPVLDSDGSYFMYSKLR
VEKKNWVERNSYSCSVVHEGLHNHHTTKSFSR*

Meditope-enabled pertuzumab:

LC:

DIQLTQSPVILSASVGDRVTITCRAGESVDIFGVGFLHWYQQRTNGSPRLLIYRASNLESG
VPSRFSGSGSRTDFTLTISSLQPEDEADYYCQQTNEDPYTFGAGTKVEIKRTVAAPSVFIF
PPSDEQLKSGAASVVCLLNNFYPREAKVQWKVDNALQSGNSQESVTEQDSKDSTYSLS
STLTLSKADYEKHKVYACEVTHQGLSSPVTKSFNRGEC*

HC:

EVQLVESGGGLVQPGGSLRLSCAASGFTFTDYTMDWVRQSPGKGLEWVADVNPNSGG
SIYNQRFKGRFTLSVDRSKNTLYLQMNSLRAEDTAIYYCARNLGDGFYAMDYWGQGTL
VTVSSASTKGPSVFPLAPSSKSTSGGTAALGCLVKDYFPEPVTVSWNSGALTSGVHTFPA

VLQSSGLYSLSSVVTVPSSSLGTQTYICNVNHKPSNTKVDKKVEPKSCDKTHTCPPCPAP
ELLGGPSVFLFPPKPKDTLMISRTPEVTCVVVDVSHEDPEVKFNWYVDGVEVHNAKTKP
REEQYNSTYRVVSVLTVLHQDWLNGKEYKCKVSNKALPAPIEKTISKAKGQPREPQVY
TLPPSRDELTKNQVSLTCLVKGFYPSDIAVEWESNGQPENNYKTTPPVLDSDGSFFLYSK
LTVDKSRWQQGNVFSCSVMHEALHNHYTQKSLSLSPGK*

Surface plasmon resonance

All measurements were fit using global fit analysis using BiaEvaluation software to minimize the effects of protein orientation on the chip surface, temperature drift, pipetting and mechanical errors. All reagents and protein samples were of highest purity and each run included a control sample with known kinetics (original meditope – CQFDLSTRRLKC). For measurements with $n<3$ the error estimate based on previous studies of meditope-Fab interactions performed in our laboratory is 10-50% [1-6]. All measurements with $n\geq3$ include standard deviation. Of note: the half-life of the complex is given by $\tau_{1/2}=k_{off}/\ln(2)$, where k_{off} is a first order reaction constant and thus independent of the analyte concentration (i.e., the units are s^{-1}).

Supplementary References

1 Donaldson, J. M. *et al.* Identification and grafting of a unique peptide-binding site in the Fab framework of monoclonal antibodies. *Proceedings of the National Academy of Sciences of the United States of America* **110**, 17456-17461, doi:10.1073/pnas.1307309110 (2013).

2 Zer, C. *et al.* Engineering a high-affinity peptide binding site into the anti-CEA mAb M5A. *Protein engineering, design & selection : PEDS*, 1-9, doi:10.1093/protein/gzx016 (2017).

3 Avery, K. N., Zer, C., Bzymek, K. P. & Williams, J. C. Development of a High Affinity, Non-covalent Biologic to Add Functionality to Fabs. *Scientific Reports* **5**, 7817, doi:10.1038/srep07817 (2015).

4 Bzymek, K. P., Ma, Y., Avery, K. A., Horne, D. A. & Williams, J. C. Cyclization strategies of meditopes: affinity and diffraction studies of meditope-Fab complexes. *Acta crystallographica. Section F, Structural biology communications* **72**, 434-442, doi:10.1107/s2053230x16007202 (2016).

5 Bzymek, K. P., Avery, K. A., Ma, Y., Horne, D. A. & Williams, J. C. Natural and non-natural amino-acid side-chain substitutions: affinity and diffraction studies of meditope-Fab complexes. *Acta crystallographica. Section F, Structural biology communications* **72**, 820-830, doi:10.1107/s2053230x16016149 (2016).

6 Bzymek, K. P., Ma, Y., Avery, K. N., Horne, D. A. & Williams, J. C. Meditope-Fab interaction: threading the hole. *Acta Crystallographica Section F* **73**, 688-694, doi:doi:10.1107/S2053230X17016272 (2017).
